# The mycotoxin deoxynivalenol activates GABAergic neurons in the reward system and inhibits feeding and maternal behaviours

**DOI:** 10.1007/s00204-020-02791-6

**Published:** 2020-05-29

**Authors:** Vivien Csikós, Petra Varró, Veronika Bódi, Szilvia Oláh, Ildikó Világi, Arpád Dobolyi

**Affiliations:** 1 MTA-ELTE Laboratory of Molecular and Systems Neurobiology, Department of Physiology and Neurobiology, Eötvös Loránd University and the Hungarian Academy of Sciences, Budapest, Hungary; 2 Department of Physiology and Neurobiology, Institute of Biology, Eötvös Loránd University, Budapest, Hungary

**Keywords:** Mycotoxin, Deoxynivalenol, c-Fos, Feeding, Maternal behaviour, Female rat

## Abstract

Deoxynivalenol (DON) or vomitoxin, is a trichothecene mycotoxin produced mainly by *Fusarium graminearum* and *culmorum.* Mycotoxins or secondary metabolic products of mold fungi are micro-pollutants, which may affect human and animal health. The neuronal and behavioural actions of DON were analysed in the present study. To address, which neurons can be affected by DON, the neuronal activation pattern following intraperitoneal injection of DON (1 mg/kg) was investigated in adult male rats and the results were confirmed in mice, too. DON-induced neuronal activation was assessed by c-Fos immunohistochemistry. DON injection resulted in profound c-Fos activation in only the elements of the reward system, such as the accumbens nucleus, the medial prefrontal cortex, and the ventral tegmental area. Further double labelling studies suggested that GABAergic neurons were activated by DON treatment. To study the behavioural relevance of this activation, we examined the effect of DON on feed intake as an example of reward-driven behaviours. Following DON injection, feed consumption was markedly reduced but returned to normal the following day suggesting an inhibitory action of DON on feed intake without forming taste-aversion. To further test how general the effect of DON on goal-directed behaviours is, its actions on maternal behaviour was also examined. Pup retrieval latencies were markedly increased by DON administration, and DON-treated mother rats spent less time with nursing suggesting reduced maternal motivation. In a supplementary control experiment, DON did not induce conditioned place preference arguing against its addictive or aversive actions. The results imply that acute uptake of the mycotoxin DON can influence the reward circuit of the brain and exert inhibitory actions on goal-directed, reward-driven behaviours. In addition, the results also suggest that DON exposure of mothers may have specific implications.

## Introduction

Mycotoxins represent a significant health challenge to animals and humans, too, as they may be digested with infected cereals, such as wheat, barley, rye, rice, and maize (Pestka 2010). Deoxynivalenol (DON) is one of the most abundant and economically most important mycotoxins. This trichothecene structured toxin is mainly produced by *Fusarium graminearum* and *culmorum*. The appearance of these *Fusarium* species is on the rise due to global warming. Numerous studies have documented that DON is heat stable. Therefore, it withstands cooking and cereal processing, which increases the risk of its occurrence in food (Hughes et al. 1999; Schothorst and van Egmond 2004; Turner et al. 2010). For this reason, DON has been implicated in mycotoxicosis. Furthermore, it was also established that DON can penetrate the blood–brain barrier (Behrens et al. 2015) and thus, directly modulate brain activity even if DON entered the brain more slowly and peaked at lower concentrations compared to other tissues, such as heart, spleen, kidney or liver (Pestka et al. 2008). A variety of different effects of DON have previously been proposed. It was shown to bind to the 60S ribosomal subunit and inhibit the biosynthesis of protein, a potential background mechanism of its cytotoxic effects. DON has negative effects on the immune system and causes intestinal inflammation (Awad et al. 2013; Pestka 2010). Other studies reported that low concentrations of DON (less than 5 mg/kg feed) stimulated the immune system while high concentrations suppressed the immune responses (Pestka 2003). Moreover, DON induced anorexia (Lebrun et al. 2015) through the brain serotonin pathways or by a direct effect on the gut microbiota (Peng et al. 2017). DON may also affect other monoamine systems, e.g. 6 weeks long DON treatment increased the dopamine and noradrenaline levels in different brain regions in mice (Al-Hazmi et al. 2015). In addition, cardiac dysfunction and transient negative effects on the autonomous nervous system were also observed in rats (Ngampongsa et al. 2011).

A possible way to explore the site of actions of DON in the brain is to examine if DON increases neuronal activation, and if yes, in which brain areas. Visualization of the immediate early gene c-Fos is a generally used and suitable marker to assess increased neuronal activity at a high resolution as the presence of c-Fos can be detected in individual cells (Herrera and Robertson 1996). c-Fos is expressed in neurons if their activity is elevated. Even if one must bear in mind that not all activated neurons show c-Fos induction, and that the threshold of c-Fos protein induction may differ between subpopulations of neurons, mapping of c-Fos expression is a useful approach to identify and investigate neuronal groups activated in response to different challenges throughout the brain (Perez-Cadahia et al. 2011). Importantly, the c-Fos technique can be used to establish the brain site of action of toxins, including DON. Indeed, some research groups have used this method to identify brain structures activated in response to DON intoxication. c-Fos activation was found in the accumbens nucleus (NAc), paraventricular nucleus of the hypothalamus, paraventricular nucleus of the thalamus, and the locus coeruleus following a low dose (100 µg/kg/day) chronic DON treatment (Faeste et al. 2019). Acute oral exposure to DON at a high dose (5 mg/kg) induced c-Fos labelling in circumventricular organs and surrounding structures (Girardet et al. 2011a). Furthermore, neurons were activated in the nucleus of the solitary tract in mice after 12.5 mg/kg acute oral DON treatment and 15% of the activated neurons were tyrosine-hydroxylase (TH)-positive (Girardet et al. 2011b). The same research group also reported c-Fos activation in the central nucleus of the amygdala and the dorsolateral division of the bed nucleus of the stria terminalis after 12.5 mg/kg DON treatment using gavage intubation needle. They also showed that DON administration decreased both meal frequency and size (Girardet et al. 2011b) suggesting nausea-induced anorexia. These experiments suggest that DON can act on the central nervous system, but the doses used in these studies were above the EU tolerable daily intake limit of DON. Therefore, we have chosen lower doses for our studies.

Reward plays a major role in different types of goal-directed behaviours, for example, feed intake, sexual activity, and maternal care (Schultz 2015). The major component of the brain reward network is the NAc, the medial prefrontal cortex (MPFC), and the ventral tegmental area (VTA) (Kelley and Berridge 2002). Dopaminergic neurons in the VTA project to the NAc and the MPFC in the mesolimbic pathway (Kardos et al. 2019; Schultz 2015). Rewarding stimuli increase the activity of dopaminergic neurons in the VTA, whereas aversive stimuli mostly inhibit their activity (Cohen et al. 2012; Matsumoto and Hikosaka 2009). The NAc has been divided into a core and a shell region, which may be implicated in different aspects of reward processing (Li et al. 2018). The GABAergic medium spiny neurons are the major projection cells in the NAc while local inhibitory GABAergic neurons are also present including those expressing parvalbumin (Yager et al. 2015). In the present study, we addressed the effect of DON of neuronal activation in the brain including the reward system. We attempted to identify the neurochemical characteristics of the activated neurons using double immunolabeling in rats and c-Fos labelling of DON treated transgenic mice whose GABA-ergic cell bodies contained green fluorescence. We also investigated the effect of DON on the feeding and maternal behaviour in the rat. For feeding, we chose a paradigm, in which the effect of a bolus injection of a drug, here DON, can be investigated (Fuller and Snoddy 1968). We chose maternal care as the other motivated behaviour to be examined, in which the NAc plays a crucial role (Olazabal et al. 2013; Salgado and Kaplitt 2015; Smith and Holland 1975).

## Materials and methods

### Animals and housing conditions

All procedures involving rats were carried out according to experimental protocols approved by the Animal Examination Ethical Council of the Animal Protection Advisory Board at Eötvös Loránd University, Budapest, and met the guidelines of the Animal Hygiene and Food Control Department, Ministry of Agriculture, Hungary. All efforts were made to minimize the number of animals used and their suffering. The animals were kept in standard laboratory conditions; the temperature was kept constant (23 ± 1 °C) in 50–60% humidity, with 12-h light–dark cycle (lights on at 6:00 AM). All experimental procedures were conducted in a separate procedure room where the animals were kept for the entire experiment. The animals were supplied with feed (SM R/M-H, 1534-00; Ssniff, Soest, Germany) and drinking water ad libitum except for rats under the fasting protocol. Two rats were housed together in a cage for the rat experiments. For the mice studies, four mice were housed together per cage. To avoid stress for mice, rats and mice cages were kept in separate rooms. A total of 43 male (310–340 g) and 48 female (200–300 g) Wistar rats (ToxiCoop, Budapest, Hungary) and 8 VGAT-IRES–Cre/GT(ROSA)26Sor_CAG/ZsGreen1 mice (VGAT-IRES-Cre mice crossed with Gt(ROSA)_CAG/ZsGreen1 mice) were used in the study. Both transgenic mouse lines were obtained from The Jackson Laboratory (Bar Harbor, Maine, United States). Female mice were 3–6 months old when mated with males with the other genotype so that all pups contained both genotypes. Therefore, genotyping after breeding was not performed in the crossed mice. It was also not needed because of the appearance of fluorescence in GABA-ergic neurons in histological sections of their brains. ZsGreen, a modified green fluorescent protein, is frequently used in histological studies of transgenic mouse models as it has bright fluorescence with high photo-stability, which is not affected by paraformaldehyde fixation and ensures excellent detail within fixed tissue (Wouters et al. 2005). In the applied mouse line, ZsGreen was expressed by the promoter of the vesicular GABA transporter (VGAT) to visualize GABAergic neurons using VGAT as a specific marker of this type of cells (McIntire et al. 1997).

### DON treatment

Deoxynivalenol (DON) was purchased from Tocris Bioscience (Bristol, United Kingdom, product number: 3976). It was dissolved in physiological saline solution before administration to the animals. DON was injected intraperitoneally (i.p.) to rats and mice in a dose of 1 mg/kg bw, while control animals received physiological saline injections.

### Tissue collection for immunolabeling

Two hours after DON (or control saline) injection, the animals were sacrificed with an intraperitoneal injection of urethane (4% dissolved in saline), 1 ml for rats, and 0.1 ml for mice. Then, the animals were transcardially perfused first with saline to remove the blood and then with 4% paraformaldehyde prepared in 0.1 M phosphate buffer (PB; pH = 7.4; Sigma, St. Louis, Missouri, United States, catalogue numbers: 71500 and S0876). Brains were removed, postfixed in 4% paraformaldehyde for a day, and then transferred to PB containing 20% sucrose for an additional day for cryoprotection. Serial free-floating coronal sections were cut at 50 µm thickness for rats and 40 µm thickness for mice with cryostat (Leica CM1520). Sections were collected in PB containing 0.1% sodium azide in parallels (5 parallels for rats and 3 for mice). The parallels were used separately for individual staining of c-Fos and c-Fos double labelling. The sections were stored at 4 °C until immunohistochemical procedures started.

### c-Fos immunolabeling in rats and mice

Every fifth 50-μm-thick free-floating coronal brain section of DON injected, and control injected rats (*n* = 6 per group) and every third 40-μm-thick free-floating coronal brain section of DON injected, and control injected mice (*n* = 4 per group) was processed for c-Fos immunohistochemistry as described previously (Olah et al. 2018). Sections were pre-treated in PB containing 0.3% hydrogen peroxide (Sigma, St. Louis, Missouri, United States, catalogue number: H1009) for 15 min for quenching of endogenous peroxidase activity. Then, the sections were incubated in PB containing 0.5% Triton X-100 (Sigma, St. Louis, Missouri, United States, catalogue number: X100) and 3% bovine serum albumin (BSA; Sigma, St. Louis, Missouri, United States, catalogue number: A3294) for 1-h. Sections were then incubated in anti-c-Fos antiserum (1:6000, Abcam, Cambridge, United Kingdom, catalogue number: ab190289) at room temperature for two nights. Following the primary antibody, the sections were incubated in biotin-conjugated anti-rabbit secondary antibody (1:800; Jackson ImmunoResearch West Grove, Pennsylvania, United States, catalogue number: 711-065-152) for 1 h and then in avidin–biotin–peroxidase complex (ABC; 1:500; Vector Laboratories, Burlingame, United States, catalogue number: PK6100) for 1 h. The labelling was visualized by nickel-2′-diaminobenzidine (DAB) peroxidase technique (Sigma, St. Louis, Missouri, United States, catalogue numbers: D5637 and N4882). Briefly, the sections were then treated with 0.06% DAB and 0.003% H2O2 in Tris hydrochloride buffer (0.05 M, pH = 8.2) containing 0.2% nickel-sulphate for 10 min. Sections were then mounted, dried, and coverslipped with Depex mounting medium (Sigma, St. Louis, Missouri, United States, catalogue number: 06522).

### Double immunolabeling in rats

Every fifth 50-μm-thick free-floating brain section from the relevant regions (NAc, VTA, MPFC) of DON injected and control injected rats was processed first for c-Fos immunohistochemistry as described earlier, except that the signal was visualized by incubation in fluorescein isothiocyanate-tyramide (FITC tyramide; 1:8000) and 0.001% hydrogen peroxide in Tris-hydrochloride buffer (0.1 M, pH = 8.0) for 6 min instead of DAB. Subsequently, the sections were incubated in mouse anti-tyrosine hydroxylase (TH) antiserum (1:5000; Chemicon, Temecula, California, United States, catalogue number: MAB5280) or mouse anti-parvalbumin antiserum (1:1000; Sigma, St. Louis, Missouri, United States, catalogue number: P3088) for overnight at room temperature, and then in Alexa 594 donkey anti-mouse (1:500, Jackson ImmunoResearch, catalogue number: 715-585-150) for 1 h. Sections were then mounted, dried, and coverslipped with Aqua-Poly/Mount (Polyscience, catalogue number: 18606).

### Double labelling of c-Fos and GABA-ergic neurons in mice

VGAT-IRES –Cre/GT(ROSA)26Sor_CAG/ZsGreen1 mice were injected with DON and control saline injection. Every third 40 μm-thick free-floating brain section from the relevant regions (NAc, VTA, MPFC) of DON injected and control injected mice was processed for c-Fos fluorescence immunohistochemistry as described earlier, except that the signal was visualized by incubation in Alexa 488 donkey anti-rabbit secondary antibody (1:500, Jackson ImmunoResearch, West Grove, Pennsylvania, United States, catalogue number: 711-545-152) for 1 h. Sections were then mounted, dried, and coverslipped with Aqua-Poly/Mount (Polyscience, Hirschberg an der Bergstrasse, Germany, catalogue number: 18606).

### Analysis and quantification of c-Fos and double labelled cell numbers in rats and mice

Brain areas were identified using the Paxinos and Watson stereotaxic atlas of the rat brain (Franklin and Paxinos 1997; Paxinos and Watson 2005). The brain areas containing Fos-immunoreactive (Fos-ir) cells were detected and captured with a light microscope equipped with epifluorescent illumination (Nikon Eclipse Ni) and a digital 2MP Slider CCD camera (Diagnostic Instruments, Sterling Heights, MI, USA) using 4–40× objectives and Spot RT3 software. The images of fluorescence double immunolabeling were taken with a confocal microscope (Zeiss LSM 800 Confocal Microscope) using 40–60× objectives. There were at least 5 z-stack images taken for each field for the analysis of double labelling at an optical thickness of 2–5 µm. Contrast and sharpness of the images were adjusted using the “levels” and “sharpness” commands in Adobe Photoshop CS 8.0. Full resolution was maintained until the photomicrographs were cropped and assembled.

The numbers of c-Fos-, and the double-labelled neuronal cell bodies were counted in brain areas where we observed a considerably high number of increased c-Fos-positive cells following DON injection. The images were coded and the person counting the cells was not aware of which experimental group the images belonged to. The numbers of c-Fos-, TH-, parvalbumin-positive or GABAergic neuronal cell bodies were counted in the coronal section where the size of the anatomical structure was the largest so that the highest number of immunolabeled cells can be observed in the brain area. In addition, cells were also counted in the previous and following Sects. (200 mm apart in the rats and 120 m apart in the mice). Thereby, the same anatomical locations were analysed in each animal.

Data from all 6 rats per group (“DON injected” and “saline-injected control” groups) and all 4 mice per group were analysed, and the density of labelled neurons was expressed as the cell number/mm^2^. The total number of Fos-ir neurons in activated regions was counted using ImageJ software, version 1.50i (ImageJ, RRID:SCR_003070, Wayne Rasband, National Institute of Health, Bethesda, MD) in photomicrographs. Statistical analysis was performed using Student’s *t* test for each brain region. All statistics were performed with Prism 6 for Windows (GraphPad Software, La Jolla, CA).

### Measurement of feeding

**Fig. 1:**
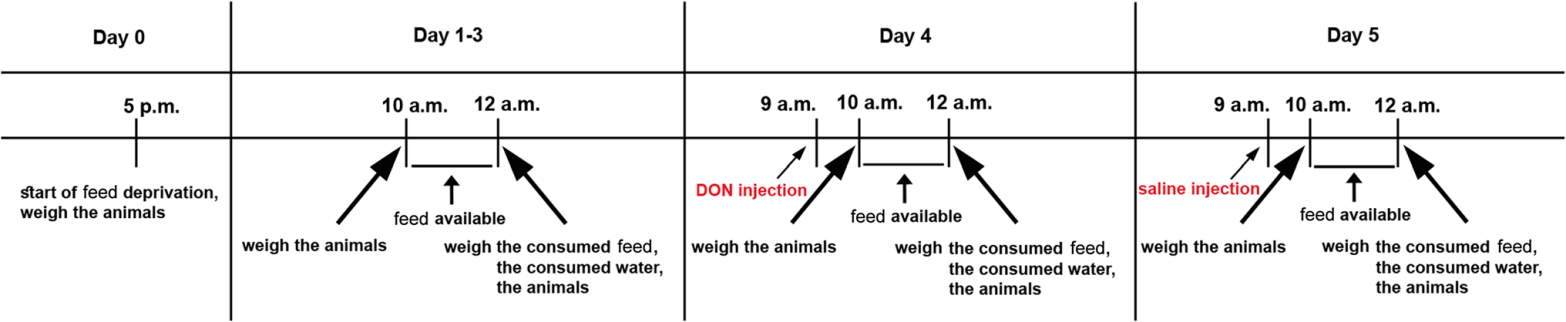
Overview of the feed intake test. The animals are deprived from the feed one day before starting the test. On days 1–3, during the habituation period, the animals can take feed only for 2 h long daily. The weight of the animals and of the consumed feed, and water are measured. On day 4, the animals are injected with DON (or saline in the control group) 1 h before the feeding period. The schedule of measurements is the same as for the habituation days. On day 5, the protocol for day 4 was performed using saline injection instead of DON to examine the return of feed intake to the control value following DON administration the previous day

The test lasted for 6 days (days 0-5), during which male and female rats (*n* = 20 males and 20 females) were kept alone in a cage. The animals were separated in the morning and were feed deprived in the evening on day zero. The experiment consisted of a training and a testing phase. The training phase was on days 1–3. On these days, the animals received feed between 10.00 to 12.00 AM. Within the 2 h, all animals had full access to feed. The testing phase consisted of days 4 and 5. The animals received i.p. DON (1 mg/kg) or saline injection at 9 AM, 1 h before feeding on the 4th day. On the 5th day, animals received a saline injection at 9 AM, 1 h before the access to feed. The amounts of consumed feed and water were measured every day before and after feeding. The weight of the animals was also measured before and after the feeding on each day to establish the effect of DON on feed intake. The protocol is summarized in Fig. 1. For a better comparison of the data, relative feed intake was calculated as the percentage of daily feed intake/body weight. The statistical analysis was carried out on this parameter with GraphPad Software using Repeated Measure 2-way ANOVA test with the type of treatment (DON vs. saline) being one parameter and the day being the repeated parameter so that to compare the maternal performance of the rat with her previous day performance.

### Measurement of maternal behaviour

Spontaneous maternal behaviour was measured as reported previously (Leko et al. 2017). For mating, 2 females and a male rat were kept together for 2 weeks. Then, pregnant females and mothers (*n* = 10) were housed individually until the end of the experiment in a separate room dedicated to the experiment. The number of pups of the mother rats was reduced to 10 immediately after parturition. The maternal behaviour of the animals was examined on postpartum days 7–8. On the first test day, animals received saline injection as control. On the second test day, the treated group of the animals received DON (i.p. 1 mg/kg) while the control group was injected with saline. The pups were removed from the mothers immediately before the injections. Pup retrieval tests were performed at 30 and 60 min after the injections. The pups were placed in corners opposite to the nest in the cage. The mothers took the pups back to the nest one by one, the timing of which was registered. The pup retrieval test took a maximum of 5 min. After the second pup retrieval test, the spontaneous maternal behaviour of the mothers was also videotaped. The videotapes were evaluated with Solomon Coder software (Version: Beta: 2016.06.26.) by an individual blind to the genotype. The time spent in the nest, the duration of suckling, kyphosis, nest building, grooming of pups, self-grooming, and exploration outside of the nest were measured.

The effect of DON on pup retrieval was assessed by the retrieval time of the 1st, 3rd, and last pup (10th). Spontaneous maternal behaviour was analysed based on the pup-associated (suckling, kyphosis, nest building, and grooming of pups) and the non-pup associated (exploration, self-grooming) behaviours. The statistical analysis was carried out with GraphPad Software using 2-way Repeated Measure ANOVA test with the day (that is treatment, as rats received saline the first day and DON on the second day) being one parameter, and the number of pups (first, third, or last) being the other parameter. Both parameters were set as repeated measures to compare the maternal performance of the rat with her previous day performance.

### Conditioned place preference test

**Fig. 2:**
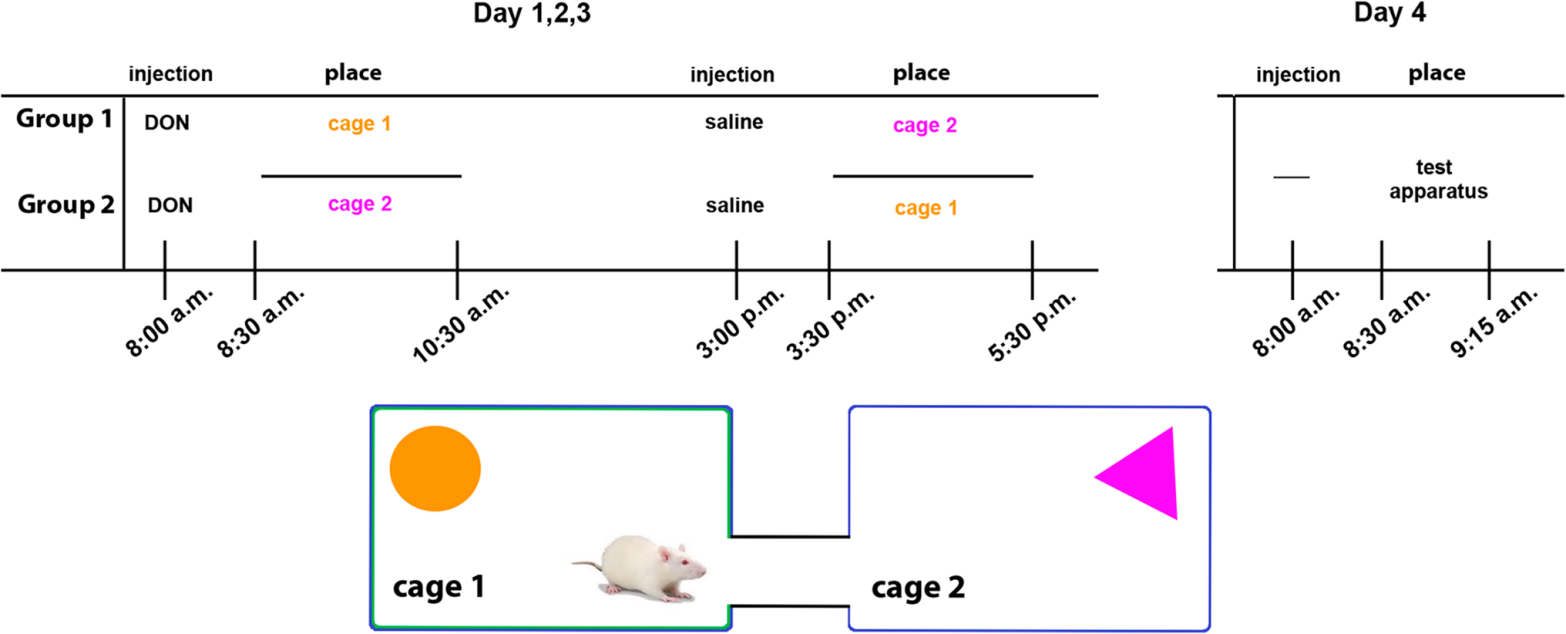
Overview of the conditioned place preference test. The upper left panel shows the protocol of establishing DON association. The 2 different cages are represented by orange and purple objects in them while the pattern on the wall of the cages was also different as shown schematically in the lower panel. The two groups differed in which type of cage was used for association with DON, that is in which cage DON was administered. The animals spent an equal amount of time in the other type of cage, too, so that familiarity with the cage would be eliminated. On the test day (day 4), the rats were placed in the test apparatus, which consisted of the 2 cages but was connected so that the animals could freely opt where to stay. Their movement was monitored without injection to test if positive or negative associations were formed for DON

To measure the addictive or aversive effect of DON, conditioned place preference was investigated as described before (Cunningham et al. 2006; Cservenak et al. 2013). Male and female rats (*n* = 11 males and 8 females) were kept alone in a cage starting 3 days before the experiment. The experiment itself took 4 days and consisted of a 3-day place conditioning while testing was performed on the 4th day (Fig. 2). For training, each animal received i.p. DON (in a dose of 1 mg/kg) or physiological saline injection in the morning and the other type of injection in the afternoon. 30 min after the injection, the animals were transferred to a special environment (new cage). There were 2 types of new cages, which both differed from the usual cage of the rats in colour and the object placed in them. The test cages were all blue, but half of them were marked with white adhesive patches. Moreover, a triangular purple or a round orange bowl was placed into the cage so that the animals could distinguish the two test cages (Fig. 2). Each animal was placed into the same type of cage following the same treatment so that the environment could be associated with the type of injection (DON vs. saline). Half of the rats received DON in one type of cage, the other half of the rats was injected with DON in the other type of cage in order to eliminate the possibility that the rats prefer one type of the cage rather than stay there altered time because of DON treatment. On the test day, the animals were placed into the test apparatus without any injection. The apparatus consisted of the 2 types of cages representing the special environment previously associated with DON or saline (Fig. 2). The 2 cages were connected with a tube so that the animals could freely choose which cage they opt to stay in. The apparatus was cleaned with 70% ethanol solution after each test to remove odour trails. The test lasted for 45 min, during which the apparatus was videotaped. The videotapes were later analysed with Solomon Coder to measure the duration of time spent in each cage.

## Results

### DON induced c-Fos activation

**Fig. 3:**
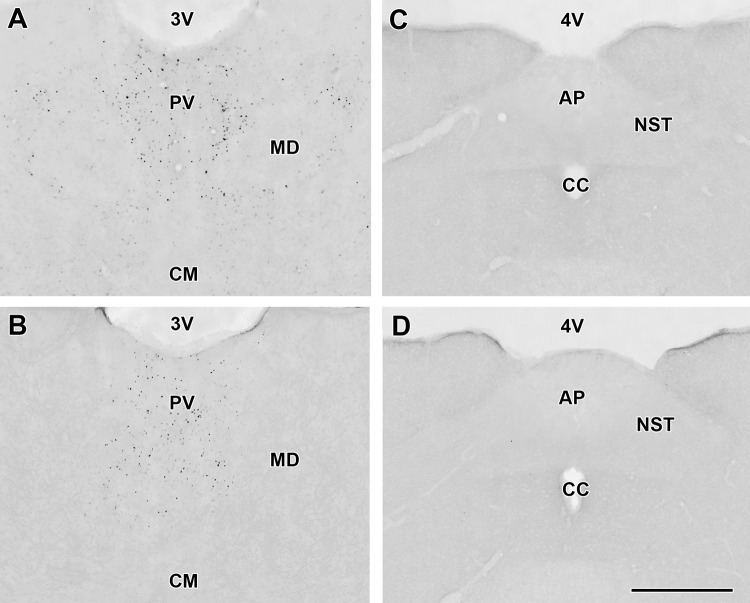
Some examples on the activation pattern of c-Fos in response to DON treatment. **a** c-Fos positive cells are shown in the paraventricular thalamic nucleus (PV) following DON administration. The abundant black dots represent the c-Fos positive cells. **b** The PV of a control (saline injected) animal contains less c-Fos positive cells than the DON injected animal, however, the density of labelled cells is still considerably high. In contrast to the PV, the area postrema (AP) and the nucleus of the solitary tract (NTS) do not express c-Fos either following DON (**c**), or following saline injection (**d**). The scale bar is 500 µm. *cc* central canal, *CM* central median nucleus, *MD* mediodorsal thalamic nucleus, *3* *V* third ventricle, *4* *V* fourth ventricle

**Fig. 4:**
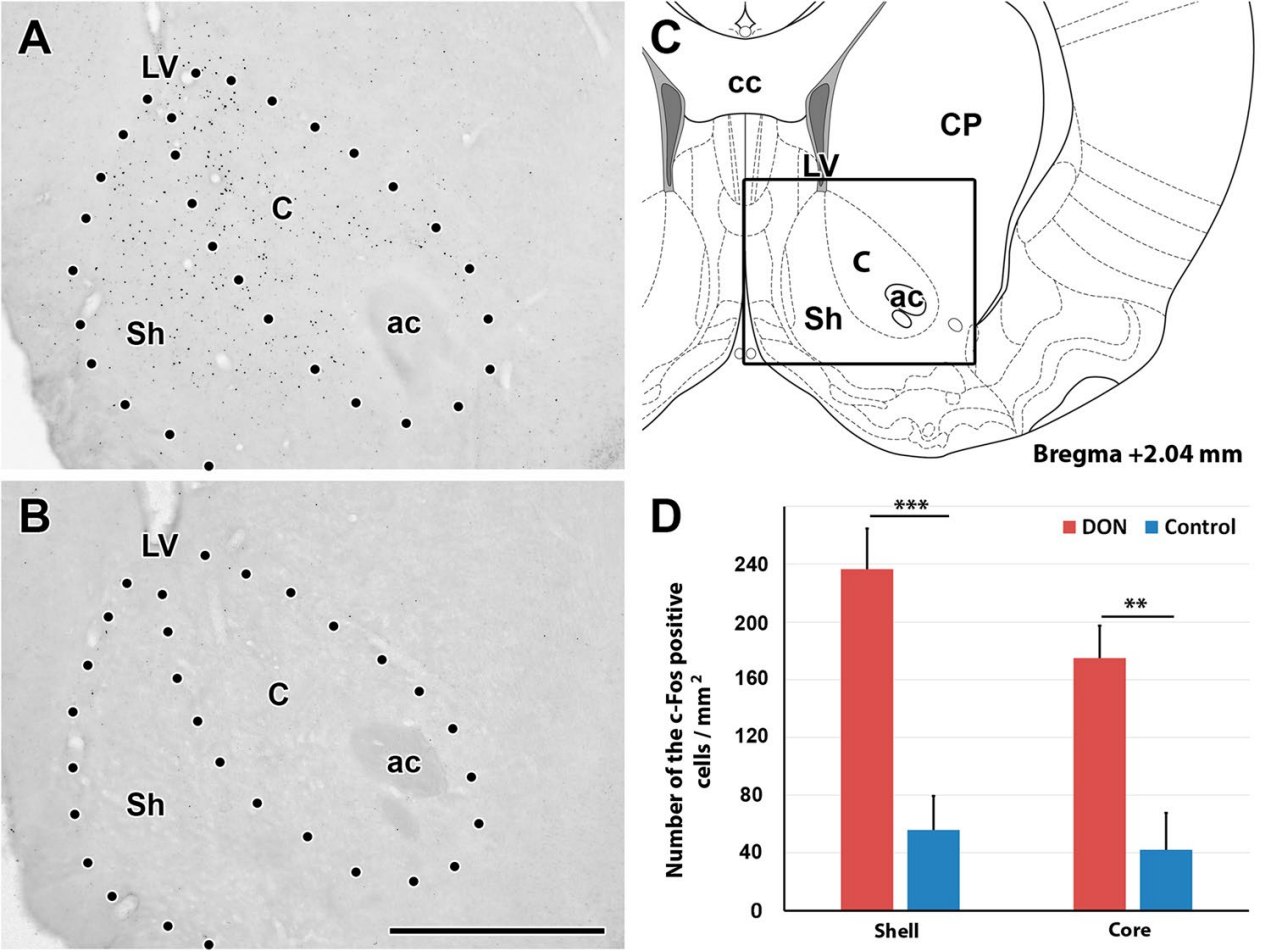
The distribution and density of c-Fos positive cells are shown in the accumbens nucleus (NAc). **a** The NAc of a DON injected animal. The black dots represent the c-Fos positive cells. They are located around the anterior commissure (ac) as well as medial to it in both the Core (C) and the Shell (Sh) region of the NAc. **b** The NAc of control (saline-injected) animal contains only few labelled cells. The scale bar is 1 mm. **c** The schematic drawing of a coronal brain section at bregma level 2.04 frames the field shown in **a**, **b**. **d** The density of c-Fos positive cells is shown separately in two different regions of the NAc. The number of c-Fos positive cells in DON injected animals are indicated with red, and the control group are signed with blue. Stars represent significant differences between DON treated and control injected animals (*p* = 0.01). *cc* corpus callosum, *CP* caudate putamen, *LV* lateral ventricle

**Fig. 5:**
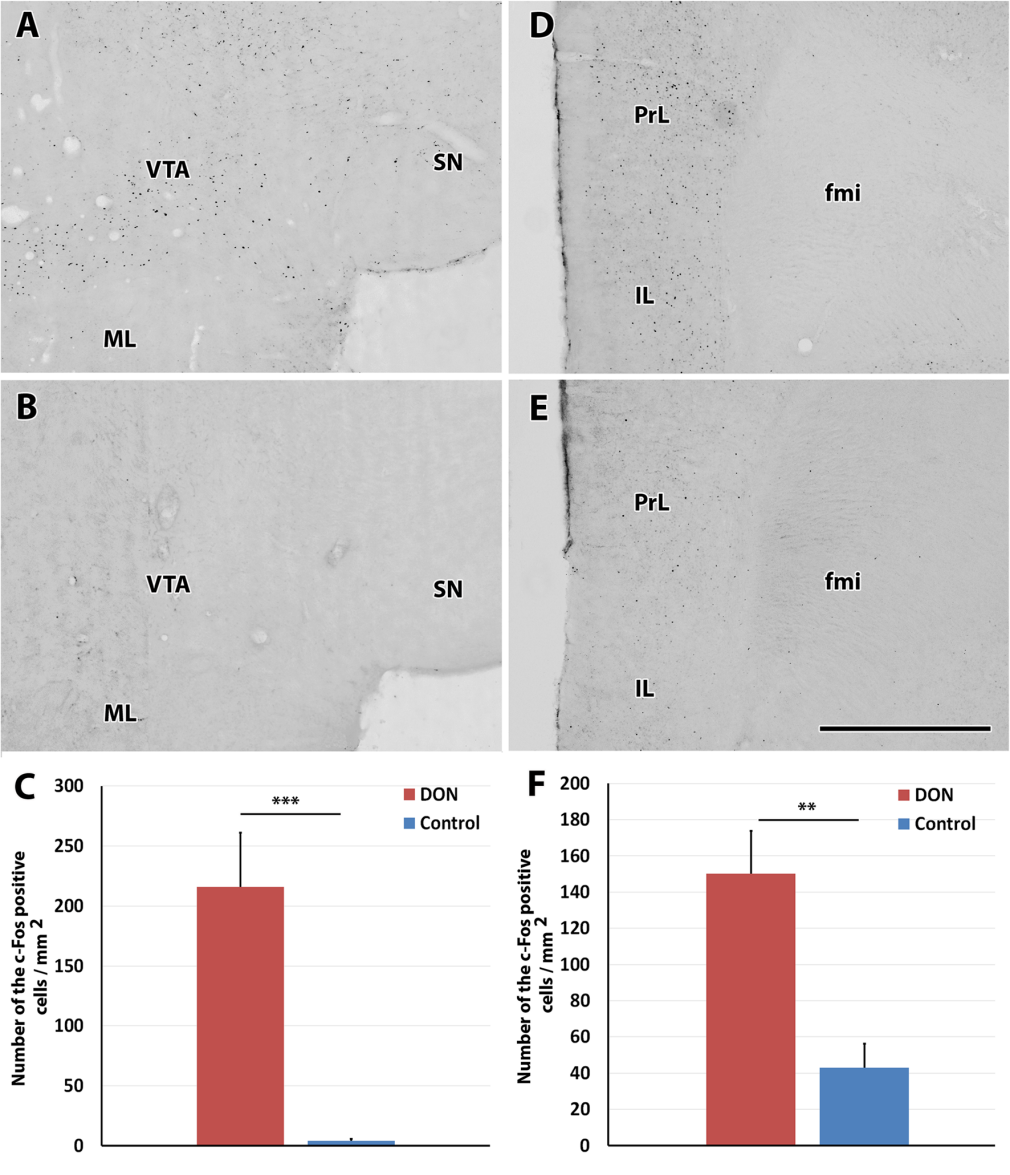
The presence and quantitative analysis of c-Fos positive cells in the ventral tegmental area and medial prefrontal cortex. **a** c-Fos positive cells (black dots) are present in the ventral tegmental area (VTA) of a DON injected animal while adjacent brain regions such as the substantia nigra (SN) and the medial mammillary nucleus (ML) do not contain c-Fos positive neurons. **b** Photomicrograph of the same field as in A of a control (saline-injected) animal. c-Fos positive neurons are not visible in the VTA. **c** The density of c-Fos positive cells in DON-treated (left, red column) and saline injected control animals (right, blue column). **d** c-Fos positive neurons are abundant in the medial prefrontal cortex of a DON injected animal. **e** The medial prefrontal cortex of a control (saline injected) animal contains only a low number of c-Fos positive neurons. **f** The density of c-Fos positive cells in each animal group in the medial prefrontal cortex. The column representing the number of c-Fos positive cells in DON injected animals is red on the left, while the column of the control group is blue on the right. *fmi* forceps minor, *IL* infralimbic cortex, *ML* medial mammillary nucleus, *Prl* prelimbic cortex, *SN* substantia nigra, *VTA* ventral tegmental area. The 4 histological panels are shown at the same magnification. The scale bar is 1 mm (colour figure online)

In response to DON treatment, c-Fos-immunolabeled cells appeared in a few brain regions while the number of labelled cells was low in these regions following control injections (Table 1). The immunolabeling was present in roundish dot-like structures suggesting nuclear localization in some brain regions but was absent in other brain regions (Fig. 3). We quantitatively evaluated the density of c-Fos positive cells in brain regions where visual mapping suggested elevated density in response to DON as compared to control saline injections and in some brain regions, which were potentially implicated in DON actions based on previous literature. Marked differences were detected in the NAc, the VTA and the MPFC (infralimbic and prelimbic cortices) of rats (Figs. 4, 5). While the density of c-Fos positive cells in NAc of the DON injected rats was 233.6 ± 39.7 on a 1 mm^2^—sized area, it was only 15.4 ± 4.4 cells/mm^2^ in control rats. For a more detailed description of the activation of the NAc, c-Fos cell density was analysed separately in the shell and core areas of NAc. At bregma level +2.04, the density of c-Fos-positive cells was 260.1 ± 30.9 cells/mm^2^in the DON injected rats and 61.7 ± 26.4 cells/mm^2^ in the control animals in the shell region. The density of c-Fos positive cells was 191.8 ± 24.2 cells/mm^2^ in the DON injected animals in the core region of the NAc while 46.3 ± 28.7 positive cells/mm^2^ were counted in the control rats in this region. The difference between the two groups was significant in both regions of the NAc (*p* < 0.01) based Student’s t test (Fig. 4). In the mice, we found 277.7 ± 50.7 c-Fos-positive cells/mm^2^ in the whole NAc after DON injection, which increased significantly from 79.3 ± 6.6 cells/mm^2^ in control mice.

**Table 1:** The number of c-Fos positive cells in a variety of different brain regions including those potentially involved in the effect of DON

Brain area	Number of c-Fos positive cells	
	Saline injected animals	DON injected animals
Cerebral cortex		
Medial prefrontal cortex		
Infalimbic cortex	+	++++
Prelimbic cortex	+	++++
Cingulate area	–	++
Dorsal peduncular cortex	–	++
Other parts of cerebral cortex	–	–
Hippocampus		
CA1 region	–	+
CA2 region	+	–
CA3 region	–	–
Dentate gyrus	–	–
Amygdala, septum		
Central nucleus	+	++
Basal nuclei	+	++
Lateral nucleus	++	+
Medial nucleus	+	++
Medial septal nucleus	–	++
Lateral septum ventral nucleus	–	++
Lateral septum intermed. nucleus	+	+
Indusium griseum		
Subfornical organ	–	–
Vascular organ of lamina terminalis	–	–
Nucleus of the diagonal band	–	–
Basal nuclei		
Caudate-Putamen	+	+
Accumbens nucleus	+	++++
Globus pallidus	+	–
Endopiriform nucleus	–	+
Claustrum	++	+
Ventral pallidum	–	+
Substantia innominata	+	++
Diencephalon		
Thalamus		
Anteromedial nucleus	+	+
Anterodorsal nucleus	+	+
Anteroventral nucleus	+	+
Paraventricular nucleus	++	++++
Midline and intralaminar nuclei	+	+
Lateral nuclei	++	++
Ventral nuclei	++	+
Reticular nucleus	+	+
Hypothalamus		
Medial preoptic area	+	+
Lateral preoptic area	+	+
Septohypothalamic nucleus	–	++
Supraoptic nucleus	+	+
Suprachiasmatic nucleus	–	–
Anterior hypothalamic area	–	+
Paraventricular nucleus	–	–
Arcuate nucleus	++	++
Lateral hypothalamic area	+	++
Ventromedial nucleus	++	++
Dorsomedial nucleus	+	++
Posterior hypothalamic area	+	++
Mamillary body	+	++
Midbrain		
Ventral tegmental area	+	++++
Periaqueductal gray	–	++
Substantia nigra	–	++
Colliculus superior	–	++
Colliculus inferior	–	–
Hindbrain		
Pons		
Pontine nuclei	–	+
Parabrachial nucleus	–	+
Superior olive	–	–
Inferior olive	–	–
Nucleus of the trapezoid body	–	+
Pontine reticular formation	–	–
Sensory trigeminal nu.	–	–
Pontine raphe nuclei	–	–
Locus coeruleus	–	+
Superior vestibular nucleus	–	+
Medial vestibular nucleus	–	–
Facial motor nucleus	–	–
Medulla oblongata and cerebellum		
Cochlear nuclei	–	+
Gigantocellular reticular nucleus	–	–
Spinal trigeminal nucleus	–	–
Medullary reticular formation	–	–
Inferior olive	–	–
Nucleus of the solitary tract	–	+
Area postrema	–	–
Cerebellar cortex	–	–
Deep cerebellar nuclei	–	–

+: the number of cells per region on 1 mm^2^ in a typical section is ≥ 50; ++: ≥ 100; +++: ≥ 150; ++++: ≥ 200

In addition to the NAc, we also observed DON-induced neuronal activation in the VTA. The number of activated neurons in the VTA was 251.3 ± 52.9/mm^2^ in DON injected (Fig. 5a) and 4.4 ± 2.2 cells/mm^2^ in control rats (Fig. 5b). The result was significant *p* < 0.01) based Student’s t test in the mouse VTA, 238.0 ± 81.6 c-Fos-positive cells/mm^2^ were counted in treated and 136.7 ± 37.5 cells/mm^2^ in control mice.

The MPFC was the third markedly activated brain region. The density of c-Fos-positive cells was 174.0 ± 28.7 cells/mm^2^ in DON injected rats (Fig. 5d) while 50.7 ± 15.4 cells/mm^2^ were c-Fos-positive in the MPFC of the control animals (Fig. 5e). In the mouse MPFC, we found 138.9 ± 6.3 activated cells/mm^2^ after DON treatment, and 32.4 ± 13.2 cells/mm^2^ at the control animals. The p-value was 0.006 with Student’s t-test.

Other brain regions were microscopically analysed, too, but no marked change was observed between the DON-treated and control animals. These data are shown in Table 1. The density of the c-Fos positive cells is the average from the analysed 6 rats.

### Phenotypic characterization of DON-activated neurons

**Fig. 6:**
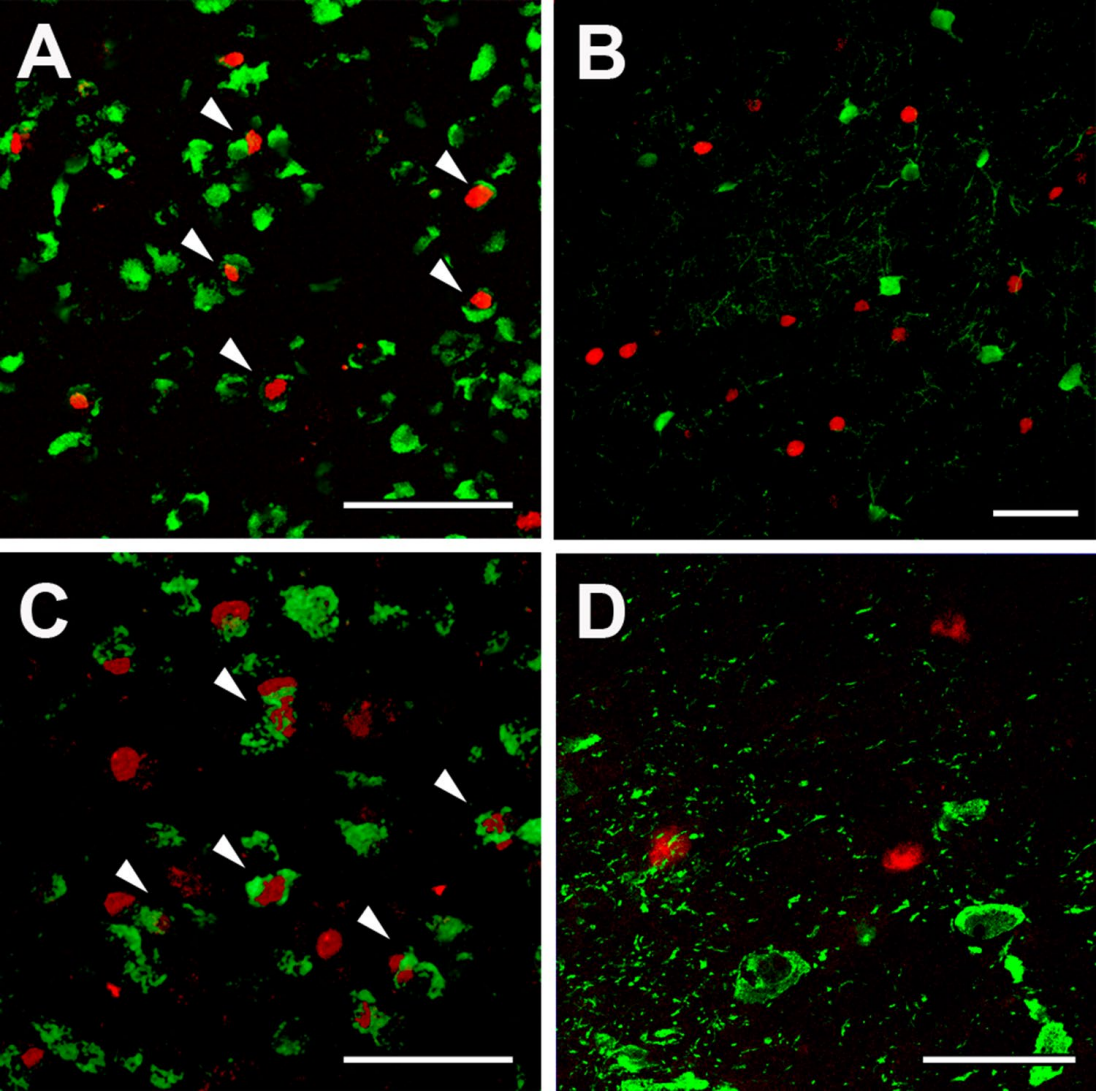
Phenotypic characterization of DON-activated neurons in the accumbens nucleus (NAc). **a** Double immunolabeling of GABAergic (green) and c-Fos positive (red) cells in the NAc. **b** Double immunolabeling of Parvalbumin (green) and c-Fos positive (red) cells in the NAc. **c** The double immunolabeling of GABAergic (green) and c-Fos positive (red) cells in the ventral tegmental area. **d** A high magnification of the ventral tegmental area about TH (green) and c-Fos (red) cells. The scale bar is 50 µm in **a**, **b** images, 25 µm in **c**, **d** images. Arrows in **c**, **d** show colocalization of GABA and c-Fos (colour figure online)

In the NAc, the number of the c-Fos-positive cells was 385.7 ± 35.3/mm^2^ in the mice while 484.9 ± 44.1 GABAergic cells/mm^2^ were counted. The number of double labelled cells was 299.8 ± 28.7 cells/mm^2^. Thus, 78% of the GABAergic cells were c-Fos-positive, and 63% of the c-Fos-positive cells were GABAergic (Fig. 6a). Parvalbumin-positive cells were also examined in the NAc where DON-activated neurons did not colocalize with parvalbumin (Fig. 6b).

In the VTA, we counted 264.5 ± 17.6 c-Fos-positive cells/mm^2^ after acute DON treatment. The number of GABAergic cells in the VTA was 579.7 ± 46.3 cells/mm^2^. The number of double labelled cells was 231.4 ± 39.7 cells/mm^2^. On average, 42% of the GABAergic cells were c-Fos-positive, and 87% of the c-Fos-positive cells were GABAergic (Fig. 6c). Dopaminergic neurons were visualized with tyrosine-hydroxylase (TH) immunohistochemistry. Labelled cell bodies were present in the VTA while fibres were observed in the NAc and MPFC. After double labelling TH and c-Fos in DON injected animals, c-Fos-positive and TH-positive neurons were both present, however, double labelled cells were not found in the VTA (Fig. 6d).

The density of the c-Fos positive cells is the average of the 4 mice analysed.

### The effect of DON on feed intake

The animals gradually increased their feed intake within the 2 h when feed was freely available for them (Table 2). Males consumed more feed than females and increased their feed intake to a larger degree from day 1 to 3.

**Table 2:** The measured weights of the rats in the beginning of the experiment and the amount of consumed feed on each day during the experiment

Number of rat	Sex	Treatment on the 4th day	Body weight (g)	Consumed food (g)				
				Day 1	Day 2	Day 3	Day 4	Day 5
1	♀	DON	237	5	9	7	6	15
2	♀	DON	204	6	7	8	5	9
3	♀	DON	209	4	6.5	6	6	10
4	♀	DON	233	5	8	8	7	11
5	♀	DON	236	2	8	10	4	13
6	♀	DON	208	9	13	20.3	5.8	19.8
7	♀	DON	170	6	9.3	10	5.1	11.5
8	♀	DON	179	4	6.3	8.3	7	12.2
9	♀	DON	175	4	7	10.5	7.5	10.8
10	♀	DON	215	14	18.5	16.5	5.8	12
11	♂	DON	224	8	12.5	15	12	18
12	♂	DON	191	7	11	16	14	18
13	♂	DON	208	6	12	16	16	18
14	♂	DON	229	11	12	16	15	17
15	♂	DON	194	9	11	12	15	15
16	♂	DON	270	11.3	12.8	13.5	9	20
17	♂	DON	277	14	19	13.8	11.8	19.2
18	♂	DON	307	10	12.5	15	10.3	14
19	♂	DON	277	7.1	6.9	12.8	7	13.7
20	♂	DON	270	7.7	11.9	17	14.5	18
21	♀	Saline	229	5	10	10	9	7.5
22	♀	Saline	192	3	9	9	8	9
23	♀	Saline	224	6	10	12	10	13
24	♀	Saline	208	3	9	8	10	10
25	♀	Saline	202	8	7	7	7	7
26	♀	Saline	254	6	11.5	14.3	12	14
27	♀	Saline	237	4.2	8.2	9	12.5	7
28	♀	Saline	245	5.6	9.7	10.5	11.6	13.2
29	♀	Saline	246	3.7	10.8	13	10	12.9
30	♀	Saline	247	8.2	10.5	12.3	11.1	13.6
31	♂	Saline	361	7	6	10	11	12
32	♂	Saline	314	8	9.5	10	12	13
33	♂	Saline	217	7	11	13	14	13
34	♂	Saline	202	6	11	13	12	10
35	♂	Saline	240	7	12	14	15	17
36	♂	Saline	267	10.7	11.6	18.3	15.2	20.1
37	♂	Saline	201	8	9.8	13.4	14.5	10.2
38	♂	Saline	235	7.3	7.8	12.3	14.9	11.9
39	♂	Saline	216	8.3	10.5	12.9	14.2	10.7
40	♂	Saline	187	8.9	8.2	9.3	8	9

DON injection affected feed consumption in both males and females as demonstrated by a reduced feed intake on the day of DON injection as compared to the control values in previous and following days. Specifically, the males consumed 14.7 ± 0.5 g feed on the 3rd control day and 17.1 ± 0.7 g on the 5th day while they consumed only 12.5 ± 0.9 g feed following DON injection (4th day). The male rats who received a saline injection on the 4th day consumed 12.6 ± 0.7 g feed on the 3rd day and 12.8 ± 1.0 g on the 5th day. After the saline injection they consumed 12.9 ± 0.7 g feed.

**Fig. 7:**
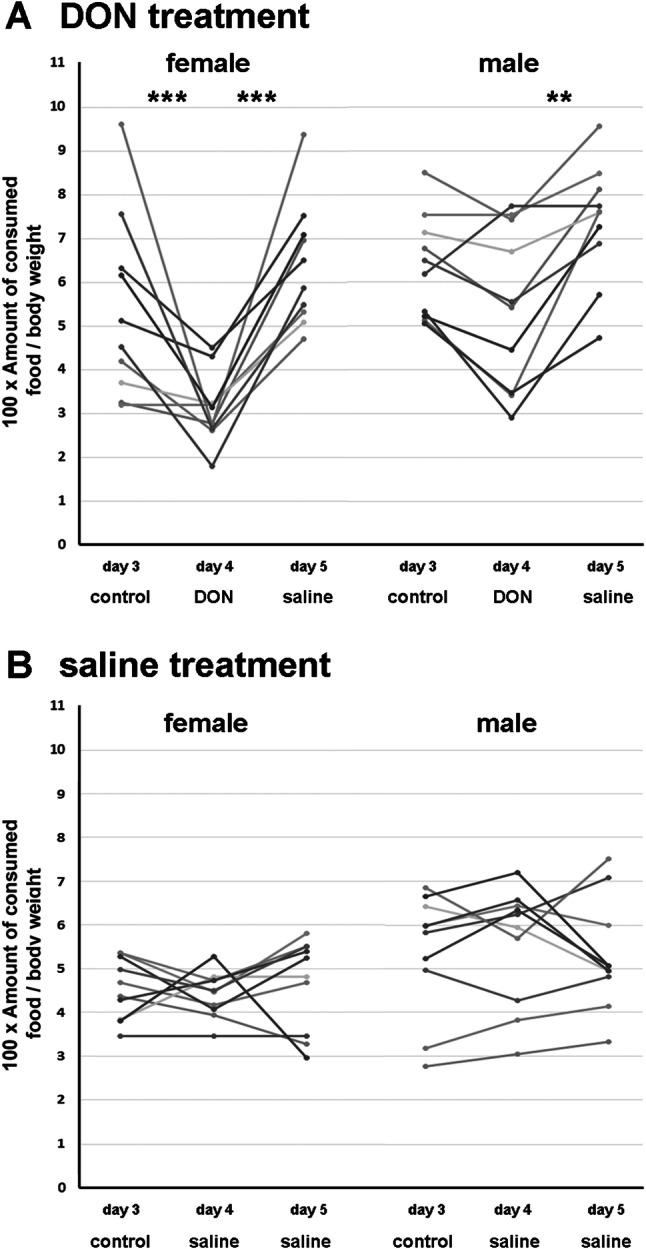
The effect of DON on feed intake in male and female after DON or saline injection. The amount of feed consumed within the 2 h feeding period relative to body weight is shown on the 3rd control day, following DON injection (**a**) or saline injection (**b**) on the 4th day, and following saline injection on the 5th day. DON reduced feed intake in both males and females on the 4th day after DON injection but not after saline injection

The females consumed 10.5 ± 1.4 g feed on the control 3rd day and 12.4 ± 1.0 g on the 5th day. 5.9 ± 0.3 g feed was consumed by them on the day of DON injection (4th day). The females consumed 10.2 ± 0.7 g feed on the 3rd and 10.5 ± 0.9 g on the 5th control day. The female rats consumed 10.1 ± 0.5 g feed after the saline injection on the 4th day. We expressed the data in relative feed intake to body weight (Fig. 7). According to the result of the 2-way Repeated Measure ANOVA test, DON treatment had a significant effect on the feed intake (F2.36) = 31.63, *p* < 0.0001). The saline injection did not have a significant effect on the feed intake (*F*(2.36) = 0.23, *p* = 0.8). Based on Tukey’s test, significant differences were found both for the males (*p* < 0.01) and the females (*p* < 0.0001) in response to DON treatment (day 4) while feed intake in the previous and following days (days 3 and 5) did not differ from each other in either sex. The saline injection did not cause changes between the days or the sexes.

Taken together, our data indicated that acute DON treatment in a dose of 1 mg/kg bw decreased the feed intake in both male and female rats.

### The effect of DON on spontaneous maternal behaviour

**Fig. 8:**
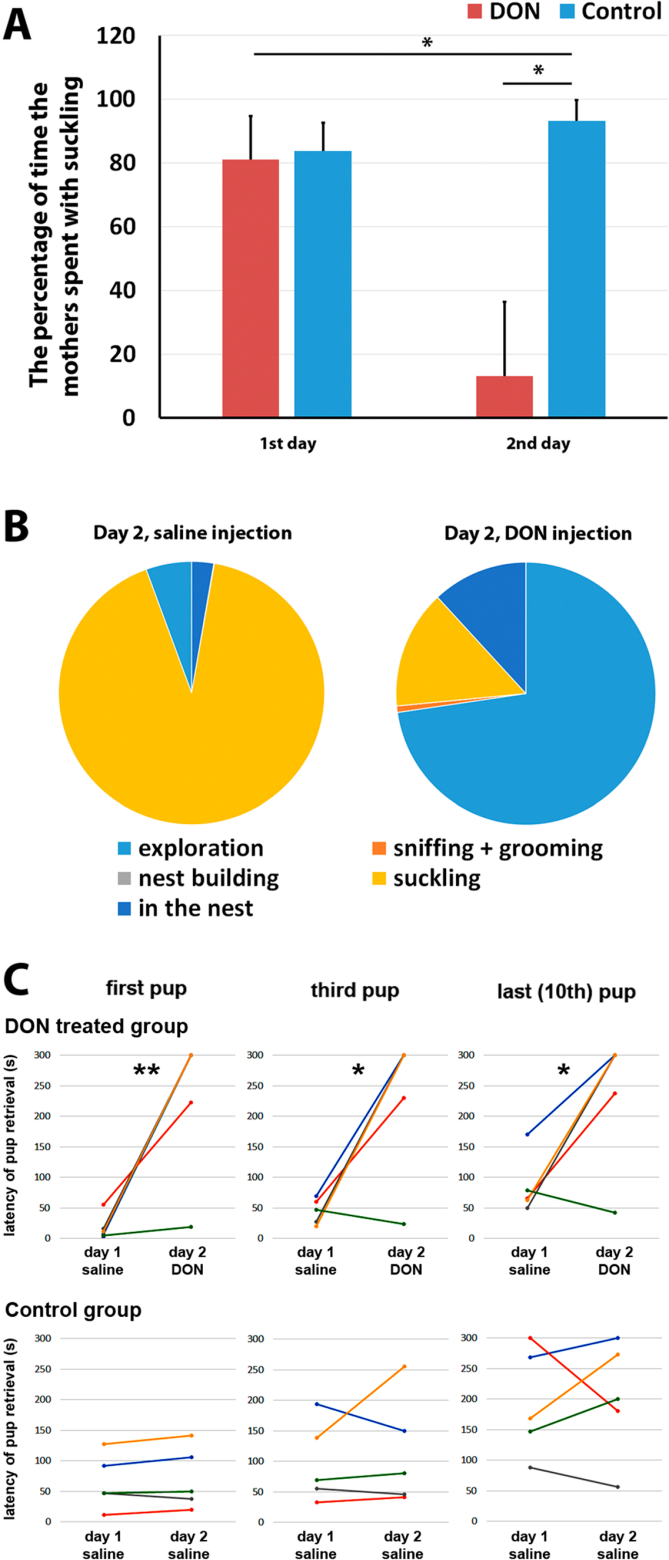
The effect of DON on maternal care. **a** The percentage of the total time the control (saline-injected) and the DON injected animals spent with suckling in the tested 60 min following the return of the pups after 1-h separation from them. On the first day, each group received saline injection and spent a similarly large percentage of their time with suckling. On the second day, saline (blue) or DON (red) was administered to the 2 groups of animals. DON injection markedly reduced the time spent with suckling as compared to the control group as well as to previous day self-control data. **b** The illustration shows how the control and DON-treated animals behaved during the test period. It is visible that the time DON injected animals saved with reduced suckling time was spent mostly with exploration. **c** The results of the pup retrieval tests (first, third and last pup) are shown in a way that data from individual animals can be appreciated. Data points of the same animals are shown with the same colour in both the DON treated and the control group. Furthermore, data points for the withdrawal time of the same number of pups (first, third or last) are connected with lines. The latency to take back the pups to the nest was significantly increased as compared to previous day control values of the same animals in response to DON injection (upper panels) whereas the latency of withdrawal was not changed between the first and second day in the group where the rats received saline injection both days (lower panel)

The control group of mothers (which received saline injection the following day) spent 81.0 ± 13.7% of their time with suckling while the treated group (which received DON injection the following day) spent 83.7 ± 8.9% with suckling on the first day. On the second day, the control group suckled the pups in 93.3 ± 6.6% of their time. In turn, the treated group spent only 13.1 ± 23.4% of their time with suckling, which is a dramatic decrease (Fig. 8a).

When mothers were not suckling, they typically explored the cage outside of the nest (DON 2nd day: 59.24%; Control 2nd day: 2.72%). In a small percentage of their time, the mothers also groomed the pups (DON: 0.65%; control: 0.08%), and they were in the nest without suckling (DON: 9.69%, control: 5.65%) (Fig. 8b). Based on Bonferroni’s multiple comparisons test, DON had a significant effect on the exploration and the suckling behaviour (*p* < 0.05).

#### Pup retrieval

When pups were placed into corners of the cage other than where the nest was located, as expected, the mothers took the pups to their mouth and carried them back to the nest. At 30 min following the injection of DON, no changes appeared in the pup retrieval behaviour of the mothers as the latencies to carry back the pups did not change as compared to the previous day latencies. In contrast, a robust effect was found at 60 min following the drug injection. On the 1st day, when both of the animal groups (DON and control) received saline injection, the pup retrieval time of the 2 groups were 18.8 ± 9.3 s and 64.7 ± 20.2 s at the 1st pup, 44.5 ± 9.3 s and 97.8 ± 29.7 s at the 3rd pup and 85.3 ± 21.6 s and 194.3 ± 39.3 s at the 10th pup, respectively. On the 2nd day, when they were administered saline or DON, respectively, the control animal group took the first pup back to the nest with a latency of 70.7 ± 22.6 s, which represents a 109.3% related to the retrieval time on the previous day. Taking the third pup to the nest needed 114.6 ± 20.2 s, which is 117.1% the retrieval time shown in the previous day value in the control animals. Carrying back the last pup (10th) to the nest, the control group took 201.9 ± 42.7 s, which is 103.9% of their time in percentage of the previous day latency. The DON injected group took the 1st pup back to the nest 228.3 ± 54.5 s, which is a 1214.4% increase compared to the previous day latencies (Fig. 8c). Following DON treatment, they retrieved the 3rd pup in 230.7 ± . 53.6 s, which is 518.2% higher than their saline day performance. They took back the last pup into the nest in 235.9 s on the second day, which was a 276.7% increase compared to their previous control day. So, the mothers, who received DON, took significantly longer to take back the pups to the nest than when they received control saline injection based on two-way repeated measure ANOVA (F (1,4) = 11.97. Sidak’ multiple comparison test further demonstrated that the effect of DON was significant on each day. In contrast, the repeated saline injection did not have a significant effect (F (1,4) = 0.2705).

### DON treatment did not induce conditioned place preference

After the 3-day training period, when the rats received daily DON injections in the DON-associated cage while control saline injections in the control cage, the animals had a free option to stay in the DON-associated or the saline-associated cage on the test day (day 4). The males spent 51.7 ± 9.6% of their time in the DON-associated cage, and 48.1 ± 3.3% of their time in the saline-associated cage. The females spent 45.5 ± 34.0% of their time in the DON-associated cage and 54.5 ± 34% of their time in the control cage. Thus, there was no significant difference between the time the animals spent in the 2 cages in either the males or females (Student’s *t* tests).

## Discussion

### Activation of the brain by DON

DON-induced c-Fos expression was described in 2 species, rats and mice. Both exhibited similar degree and distribution of c-Fos immunoreactivity following acute intraperitoneal injection of DON (1 mg/kg bw) resulted in c-Fos labelling 2 h after its administration. In contrast, the number of c-Fos positive cells remained low following control injections suggesting specific drug actions in response to DON administration. It is known that the appearance of c-Fos protein takes about an hour following stimulation of neurons, after which c-Fos remains in the cell for a few hours (Herrera and Robertson 1996). So, DON may have reached and affected its targets within an hour. Previous data suggest that the penetration of DON to the brain is species dependent (Chen et al. 2017; Payros et al. 2016). DON concentrations in the brain reach a maximum after 5–10 min in sheep and 30–60 min in swine (Pestka 2008). In line with these data, the effect of DON on pup retrieval was not seen after 30 min but only after 60 min following i.p. administration of the drug.

Despite the systemic administration of DON, only a few brain regions demonstrated c-Fos expression. Based on the characteristics of the c-Fos technique, it is possible that some activated neurons remained unrevealed following DON injection as some neurons can be activated without the appearance of c-Fos protein (Perez-Cadahia et al. 2011). Still, the finding that most brain regions did not contain c-Fos immunoreactivity argues against a non-specific action of DON, e.g. a general neurotoxic action via the previously revealed ribosomal mechanism (Pestka 2010) to evoke c-Fos activity. Since neither sensory nor circumventricular centres showed activation by DON in our study, it is likely that DON exerted a direct effect on the c-Fos positive neurons rather than activating them from the periphery through indirect neuronal pathways, e.g. via its effect on the immune system or the intestine (Pestka 2010). DON is known to penetrate the blood–brain barrier (Behrens et al. 2015; Payros et al. 2016; Pestka et al. 2008), which allows a direct effect on its brain targets. The specificity of DON action is also supported by the finding that even within the affected brain regions, only a subset of neurons was activated. Interestingly, the activated neurons were mainly located in brain regions belonging to the reward system. Previously, the activation of only some of these brain areas, such as the accumbens nucleus was reported in response to prolonged application of DON in low dose (100 µg/kg bw) or higher acute dose (12.5 mg/kg bw) (Faeste et al. 2019). We demonstrated that 1 mg/kg bw acute injection of DON can also evoke activation of the NAc and that the increase in the neuronal activity takes place in both the core and the shell subdivisions of the nucleus. We also found similarities with previous c-Fos studies in the paraventricular thalamic nucleus reported to be activated by low dose chronic and high dose acute DON (Faeste et al. 2019). The paraventricular thalamic nucleus was also activated in a previous pig study (Gaige et al. 2013) after 1 mg/kg bw per os DON application. A substantial amount of c-Fos positive cells were found in the paraventricular thalamic nucleus following saline injection, too. It could be a consequence of the stress factor of the injection itself although it is also possible that the paraventricular thalamic nucleus contains c-Fos positive neurons in the absence of experimental stimulus. Furthermore, the number of c-Fos positive neurons seemed to be slightly increased following acute DON injection. However, the extent of the increase did not seem to be as large as in the accumbens nucleus. In addition, the present study revealed additional brain sites of activation, such as the medial prefrontal cortex and the ventral tegmental area, and thus, demonstrated a different activation pattern by acute DON administration. Our findings were similar to the pigs in some other brain regions, too, including the amygdala, and the lateral hypothalamic area. In turn, the area postrema and the nucleus of the solitary tract, brain sites involved in conditioned taste aversion, and vomiting previously reported to be activated by DON in the pigs (Gaige et al. 2013) were not affected in the present study. These differences may be explained by the lower concentration of DON applied in our study, the different mechanisms between activation following acute and chronic administration, and species differences, particularly that pigs are more sensitive to the toxin (Andretta et al. 2012).

### Behavioural consequences of acute DON injection

The reward system, which was activated by DON, plays an outstanding role in the control of goal-directed behaviours (Dulac et al. 2014), so the effect of DON on two such behaviours was investigated. In the applied feeding protocol, daily feed intake is confined to 2 h, which allows the examination of the effect of DON injection on feed intake as the feeding pattern is predictable due to the restricted availability of the feed (Fuller and Snoddy 1968) when DON concentration is the maximal. Chronic DON administration has been reported to inhibit feed intake in different species (Wu et al. 2014) including the pig (Pierron et al. 2016; Wu et al. 2014). Here, we demonstrated an inhibitory effect of acute DON injection using the restricted feed availability protocol in the rat. We also provided evidence that DON was not aversive to the animals since it did not create a negative place preference. If the toxin-induced a taste aversion in the rats, the cage with saline would be chosen. c-Fos immunoreactivity following an acute high-dose DON administration showed increased c-Fos expression in the anorexigenic circuit (Faeste et al. 2019). The lack of c-Fos activation in the dorsal vagal complex, a vomiting centre including the circumventricular organ area postrema, the nucleus of the solitary tract, and the motor nucleus of the vagal nerve argue against nausea-inducing action of DON at the low concentration applied throughout our study. Most importantly, however, the feed intake was normal a day following DON administration suggesting that conditioned taste aversion did not take place and DON inhibits feeding behaviour probably via reducing the perceived reward value of the feed. This conclusion is also supported by the finding that (1) the control animals did not show any changes in their feed intake and (2) no place preference was induced by DON administration. In a previous study, the weight reduction of female Swiss mice was significant by oral administration of 45 μg DON/kg bw/day for 7 days (Kouadio et al. 2013), which is consistent with our findings on reduced feed intake. Therefore, the effect of DON on another, this time female-specific reward-driven behaviour, maternal behaviour was also examined. DON exhibited a dramatic effect on spontaneous maternal behaviour as the time spent with suckling was markedly reduced in mother rats after DON injection. Furthermore, DON was also strongly inhibitory in an induced maternal behaviour, the pup retrieval, which is the most often used test of maternal motivation (Bridges 2015). The increased pup retrieval latencies suggest that DON reduces maternal motivation, which may have been the underlying reason of the reduced suckling behaviour, too. The maternal behavioural effects of DON have not been investigated previously. However, DON showed limited effect of sow health and production despite a reduction in feed consumption (Sayyari et al. 2018). Apart from species differences, it is possible that raising a piglet in a farm does not represent a challenging environment for maternal motivations, therefore, such an effect of DON could have stayed hidden in the absence of specific tests on it.

### Potential mechanisms of the behavioural actions

Since two different reward-driven behaviours were affected by DON treatment, and neurons in the reward system of the brain were activated, it seems likely that general reward-related rather than behaviour-specific brain mechanisms are involved in the effect of DON. Even though neurons in the NAc were activated by DON, its addictive actions are not expected as its administration did not induce place preference. We used tyrosine hydroxylase (TH) as a marker of dopaminergic neurons (Molinoff and Axelrod 1971) and found the same distribution of TH as described previously (Bjorklund and Dunnett 2007). However, dopaminergic neurons were not activated based on double labelling experiments. Instead, some GABAergic neurons showed c-Fos expression suggesting that they may mediate the actions of DON on the reward-driven behaviours. The reduced motivation may actually be related to decreased dopamine level, which has been reported following acute low dose DON injection (Bonnet et al. 2012; Prelusky et al. 1992).

It is also noteworthy to mention that we described a unique way of specifically activating some GABAergic neurons without dopaminergic activation in the reward system. Importantly, GABAergic neurons in other brain regions are not affected, which results in an experimental tool potentially useful for further studies investigating the reward system.

## Conclusions

While the actions of DON on peripheral organs and tissues is well documented, data on its effects in the brain are less abundant. In the present study, we examined the potential neurotoxicity of DON by investigating the neuronal activation pattern following its intraperitoneal injection to adult male rats at a dose of 1 mg/kg bw. Activation was particularly high in reward centres. In contrast, we did not find neuronal activation in vomiting centres or dependency to the saline injection into the conditioned place preference test. Furthermore, DON did not evoke taste aversion. In turn, we detected reduced motivated behaviours, both feeding and maternal behaviours following DON injection suggesting that DON may have an inhibitory effect on motivated behaviours in addition to its previously reported toxic actions. The more profound effect of DON on feed intake in females, as well as the dramatic effect of DON on the maternal behaviours suggest that the drug might have sexually dimorphic action: DON exposure may have more serious consequences in females. The results also point to the importance of the reproductive status regarding the effects of DON. It is typically not investigated for DON toxicology, but the toxin may have a specific effect on mothers. Therefore, special attention should be given to DON intoxication during pregnancy and the postpartum period.
